# Reactive oxygen species coordinate the transcriptional responses to iron availability in Arabidopsis

**DOI:** 10.1093/jxb/eraa522

**Published:** 2020-11-07

**Authors:** Claudia von der Mark, Rumen Ivanov, Monique Eutebach, Veronica G Maurino, Petra Bauer, Tzvetina Brumbarova

**Affiliations:** 1 Institute of Botany, Heinrich Heine University, Universitätsstr. 1, D-40225 Düsseldorf, Germany; 2 Group of Plant Vascular Development, Swiss Federal Institute of Technology (ETH) Zurich, CH-8092 Zurich, Switzerland; 3 Department of Molecular Plant Physiology, Institute of Molecular Physiology and Biotechnology of Plants, University of Bonn, Kirschalle 1, D-53115 Bonn, Germany; 4 Cluster of Excellence on Plant Science (CEPLAS), Heinrich Heine University, D-40225 Düsseldorf, Germany; 5 University of Birmingham, UK

**Keywords:** bHLH, catalase, FIT, H_2_O_2_, iron uptake, ROS

## Abstract

Reactive oxygen species play a central role in the regulation of plant responses to environmental stress. Under prolonged iron (Fe) deficiency, increased levels of hydrogen peroxide (H_2_O_2_) initiate signaling events, resulting in the attenuation of Fe acquisition through the inhibition of FER-LIKE IRON DEFICIENCY-INDUCED TRANSCRIPTION FACTOR (FIT). As this H_2_O_2_ increase occurs in a FIT-dependent manner, our aim was to understand the processes involved in maintaining H_2_O_2_ levels under prolonged Fe deficiency and the role of FIT. We identified the *CAT2* gene, encoding one of the three Arabidopsis catalase isoforms, as regulated by FIT. CAT2 loss-of-function plants displayed severe susceptibility to Fe deficiency and greatly increased H_2_O_2_ levels in roots. Analysis of the Fe homeostasis transcription cascade revealed that H_2_O_2_ influences the gene expression of downstream regulators *FIT*, *BHLH* genes of group Ib, and *POPEYE* (*PYE*); however, H_2_O_2_ did not affect their upstream regulators, such as *BHLH104* and *ILR3*. Our data shows that FIT and CAT2 participate in a regulatory loop between H_2_O_2_ and prolonged Fe deficiency.

## Teaser

We demonstrate that CAT2 modulates root H_2_O_2_ levels and forms a regulatory loop with the central Fe acquisition transcription factor FIT to influence Arabidopsis response to prolonged Fe deficiency.

## Introduction

Plants are constantly confronted with a varying array of stress conditions to which they need to respond and adapt in order to survive. Aerobic metabolic processes, such as photosynthesis and respiration, as well as chemical reactions in the cell, lead to the production of reactive oxygen species (ROS), the most prominent of them being superoxide (O_2_˙ ^−^), hydrogen peroxide (H_2_O_2_), hydroxyl radical (OH·), and singlet oxygen (^1^O_2_). ROS can be produced in different cellular compartments and have different reactivity and stability. They can increase in amount under stress conditions and damage proteins, nucleic acids, and lipids, eventually triggering cell death. On the other hand, controlled and spatially defined ROS production can trigger signaling events and initiate or contribute to specific plant stress responses (Noctor and Foyer, 2016; Mittler, 2017; Czarnocka and Karpinski, 2018; Smirnoff and Arnaud, 2019). To maintain ROS homeostasis, plants employ ROS-scavenging enzymes, such as catalases (CATs), superoxide dismutases, ascorbate peroxidases, glutathione peroxidases, and peroxiredoxins (Mittler *et al.*, 2004; Inupakutika *et al.*, 2016; Khedia *et al.*, 2019). *Arabidopsis thaliana* (Arabidopsis) has three catalase genes, *CAT1*, *CAT2*, and *CAT3*, whose products dismutate H_2_O_2_ to H_2_O and O_2_, with varying contributions of the three proteins in the different plant organs (Mhamdi *et al.*, 2010). *CAT1* is mainly expressed in pollen and seeds, *CAT2* in photosynthetic tissues, roots, and seeds, and *CAT3* in leaves and vascular tissues (Frugoli *et al.*, 1996; McClung, 1997; Mhamdi *et al.*, 2010). The differential contribution and partial functional redundancy of the three catalases in different organs has been recently studied (Su *et al.*, 2018; Yang *et al.*, 2019). CAT loss-of-function mutants have been successfully employed as a model system to study the role of ROS in abiotic and biotic stress responses (Mhamdi *et al.*, 2010; Sewelam *et al.*, 2014; Schmidt *et al.*, 2020).

An abiotic stress with a profound impact on plant health and survival is the lack of sufficient iron (Fe). Fe deficiency-induced anemia affects billions of people worldwide, necessitating the study and deeper understanding of the mechanisms that plants utilize to acquire Fe from the soil, in order to combat Fe malnutrition through the development of Fe-fortified crops (Naranjo-Arcos and Bauer, 2016). Most flowering plants, except members of the *Poaceae* family, acquire Fe by employing a reduction-based mechanism, involving the function of a root surface ferric reductase and a ZIP-family bivalent metal transporter. In Arabidopsis, the Fe reductase activity is carried by the FERRIC REDUCTASE-OXIDASE2 (FRO2) protein and transport is achieved through the action of the IRON-REGULATED TRANSPORTER1 (IRT1), these being encoded by genes up-regulated under Fe deficiency (Ivanov *et al.*, 2012; Kobayashi and Nishizawa, 2012; Brumbarova *et al.*, 2015). Transcriptionally, Fe acquisition is controlled by a series of regulatory events representing a hierarchical cascade of mainly basic helix–loop–helix (bHLH) family transcription factors (Gao *et al.*, 2019). Gene co-expression analysis defines different Fe-related regulatory modules (Ivanov *et al.*, 2012; Brumbarova and Ivanov, 2019; Schwarz and Bauer, 2020). One module consists of genes involved in the acquisition of Fe, such as *FRO2* and *IRT1*, regulated by FER-LIKE IRON DEFICIENCY-INDUCED TRANSCRIPTION FACTOR (FIT). Another module comprises genes with functions in Fe homeostasis, regulated by and co-expressed with POPEYE (PYE) (Long *et al.*, 2010). A third module contains mainly Fe storage-related genes, such as *FERRITIN* genes, also controlled by IAA-LEUCINE RESISTANT3 (ILR3) and bHLH121/UPSTREAM REGULATOR OF IRT1 (URI) (Samira *et al.*, 2018; Brumbarova and Ivanov, 2019; Kim *et al.*, 2019; Tissot *et al.*, 2019; Gao *et al.*, 2020*a*, *b*; Lei *et al.*, 2020). At the same time, ILR3, together with URI, is involved in the regulation of *PYE* and the bHLH subgroup Ib genes *BHLH038*, *BHLH039*, *BHLH100*, and *BHLH101* (Kim *et al.*, 2019; Gao *et al.*, 2020*a*; Lei *et al.*, 2020). The subgroup Ib bHLHs interact with FIT to regulate its expression, as well as the expression of *FRO2* and *IRT1* (Wang *et al.*, 2007; Yuan *et al.*, 2008; Wang *et al.*, 2013; Trofimov *et al.*, 2019). Among Ib bHLHs, *BHLH039* is a robust Fe deficiency marker gene up-regulated by Fe deficiency (Wang *et al.*, 2007; Ivanov *et al.*, 2012). Its expression is not regulated by FIT; however, when FIT is lacking, *BHLH039* undergoes additional up-regulation, following a strong Fe deficiency signal in the plant up-regulating the bHLH cascade (Wang *et al.*, 2007; Schwarz and Bauer, 2020). The result of this complex interplay of regulators is the dynamic transcriptional response ensuring the balanced acquisition, redistribution, and safe storage of Fe.

With the help of hormonal signaling, the Fe acquisition domain is dynamically regulated not only in time but also in space. Thus, the expression domain of *IRT1* is adjusted around the early differentiation zone of the root according to the Fe requirements of the plant through crosstalk between the phytohormones ethylene and auxin (Blum *et al.*, 2014). ROS, and specifically H_2_O_2_, were previously shown to influence the activity of FIT through an interaction with the oxidative stress-responsive transcription factor ZINC FINGER OF ARABIDOPSIS THALIANA12 (ZAT12) (Le *et al.*, 2016). The interaction between the two occurs under prolonged Fe deficiency, leading to FIT inactivation and attenuation of the Fe deficiency response. The specific increase in H_2_O_2_ observed under these conditions was FIT dependent and a central prerequisite for the observed major switch in the response strategy of the plant (Brumbarova *et al.*, 2016*b*; Le *et al.*, 2016, 2019; Brumbarova and Ivanov, 2018).

A question that remains open is which processes are involved in the maintenance of the increased H_2_O_2_ levels under prolonged Fe deficiency and in which way FIT participates in this regulation. Here, we show through histochemical co-staining of H_2_O_2_ and O_2_˙ ^−^ in the root that lack of functional FIT leads to spatially deregulated ROS distribution under prolonged Fe deficiency. FIT is involved in the transcriptional regulation of *CAT* genes, specifically *CAT2*. CAT2 loss-of-function mutants display severely hampered Fe deficiency responses. CAT2 is a major contributor to the H_2_O_2_-scavenging capacity of roots under prolonged Fe deficiency. Lack of CAT2 leads to a marked increase of H_2_O_2_ in root hair cells of the early differentiation zone. Gene expression analysis showed that CAT2 activity affects the regulation of *PYE* and *FIT*, together with genes from their target modules. At the same time, their upstream regulators ILR3 and bHLH104 are not affected transcriptionally by the lack of CAT2, suggesting post-translational effects of CAT2 and H_2_O_2_ on ILR3 and bHLH104 activity. Our data suggest the existence of a FIT- and CAT2-mediated feedback loop controlling the upstream regulators of Fe uptake and homeostasis under prolonged Fe deficiency.

## Materials and methods

### Plant material and growth conditions

Arabidopsis wild-type Col-0, the FIT loss-of-function *fit-3* mutant (GABI_108C10, Jakoby *et al.*, 2004), and the CAT2 loss-of-function *cat2-1* mutant SALK_076998, N576998, named *cat2-2* in Queval *et al.* (2007) and later renamed *cat2-1* as in Bueso *et al.* (2007), were used in this study. T-DNA insertion lines were in the Col-0 genetic background. Plants were grown upright for 10 d on half-strength Hoagland medium agar plates (Jakoby *et al.*, 2004) either supplemented with 50 µM FeNaEDTA (Fe-sufficient condition) or without Fe (Fe-deficient condition).

### Root length measurement

Images of plants grown upright on half-strength Hoagland medium agar plates were acquired and root lengths were measured using the JMicroVision software, version 1.2.7 (http://www.jmicrovision.com). Following spatial calibration, length measurements were made of freehand drawn lines covering the root on the image. The obtained lengths were averaged, and the SD was calculated.

### Fe reductase activity measurement

The activity of the root surface ferric reductase was measured as described in Le *et al.* (2016). Briefly, roots were rinsed in 100 mM Ca(NO_3_)_2_ solution and incubated in a solution containing 100 μM FeNaEDTA and 300 μM ferrozine for 1 h at room temperature in the dark. Enzymatic activity was calculated using the ferrozine molar extinction coefficient of 28.6 mM^−1^ cm^−1^. The experiment was performed in six replicates. Each replicate comprised a pool of five seedlings. The obtained data were normalized to the sum of the root length for each replicate. Fe reductase activity of the six replicates per condition was averaged and the SD was calculated.

### Photosynthetic pigment measurement

For plant pigment extraction, pre-weighed rosettes of 10-day-old seedlings were shock-frozen in liquid N_2_ prior to tissue rupture using 1.4 mm ceramic beads and a Precellys 24 tissue homogenizer (Bertin Instruments) three times for 20 s, at 6500 rpm. Samples were frozen in between homogenization steps in liquid N_2_. Pigment was extracted adding 500 µl of pure acetone immediately after grinding, followed by a centrifugation step (3 min, 13 000 rpm). The extraction was repeated once. The supernatant was collected and absorption was measured at 646, 663, and 750 nm in a 96-well plate reader (Infinite 200 PRO, Tecan). Concentrations (c) of Chl *a*, Chl *b*, and carotenoids (in μg ml^–1^) were calculated as described in Lichtenthaler and Wellburn (1983):

$$\begin{array}{l} c\left(Chl\;a\right)=11.75*E_{max}662-2.35*E_{max}645 \end{array}$$

$$\begin{array}{l} c\left(Chl\;b\right)=18.61*E_{max}645\;-3.96*E_{max}662 \end{array}$$

$$\begin{array}{ll} & c\left(carotenoids\right)\\ & =\left(1000*E_{max}470-2.27*c\left(Chl\;a\right)-81.4*c\left(Chl\;b\right)\right)/227,\\ & \;in\;\left[\mu g*ml^{-1}\right]. \end{array}$$

Data were normalized to the sample fresh weight to obtain the pigment content of the rosette. Six independent replicates per condition were measured, averaged, and the SD was calculated.

### Catalase activity measurement

Catalase activity was measured following the protocol described in Noctor *et al.* (2016) with some modifications, as follows. Total root and shoot extracts were prepared by grinding the frozen tissue using 1.4 mm ceramic beads and a Precellys 24 tissue homogenizer (Bertin Instruments) three times for 20 s, at 6500 rpm. The powder was homogenized in 0.1 M phosphate buffer, 1 mM EDTA (pH 7.5). The buffer volume was adjusted according to the fresh weight of the samples, so that 10 μl of buffer were added to each 1 mg of sample. The suspension was centrifuged for 10 min at 4 °C, at 13 000 rpm. H_2_O_2_ at 40 mM was added to 180 μl of each extract. The samples were immediately placed in a 96-well plate reader (Infinite 200 PRO, Tecan) and the disappearance of H_2_O_2_ absorption at 240 nm was recorded over time. The data from the first 1 min of the reaction were used to calculate catalase activity, applying the H_2_O_2_ molar extinction coefficient of 40 mM^−1^ cm^−1^. The catalase activity for three independent replicates per condition was measured, averaged, and the SD was calculated.

### H_2_O_2_ content measurement

H_2_O_2_ in root samples was measured using the Amplex Red H_2_O_2_–peroxidase assay kit (Molecular Probes) as described in detail in Brumbarova *et al.* (2016*a*). The H_2_O_2_ content values for three independent replicates per condition were measured, averaged, and the SD was calculated.

### Seed Fe content measurement

The Fe content of seeds was measured using a colorimetric assay, adapted for plant seeds from the protocol of Tamarit *et al.* (2006). Seeds were dried overnight at 100 °C, ground in an agate mortar (Merck), and weighed. A 10–15 mg aliquot of material was resuspended in 500 µl of 3% HNO_3_ and incubated for 16 h at 100 °C, followed by centrifugation for 5 min at 12 000 rpm. Then 400 µl of 3% HNO_3_ containing either FeNaEDTA standard or seed material were mixed with 160 µl of sodium ascorbate (38 mg ml^–1^), 320 µl of Fe chelator BPDS (1.7 mg ml^–1^), and 126 µl ammonium acetate solution (diluted 1:3 from saturated solution). The specific absorbance of the Fe–chelator complex was recorded at 535 nm after 5 min, together with a reference measurement at 680 nm for non-specific absorbance, using a 96-well plate reader (Infinite 200 PRO, Tecan). The obtained Fe concentration was normalized to the dry weight of the sample. The Fe content values for three independent replicates per genotype were obtained, averaged, and the SD was calculated.

### Gene expression analysis by quantitative reverse transcription–PCR (RT–qPCR)

Gene expression analysis was performed as described by Gratz *et al.* (2019). In brief, total RNA was prepared from roots of Arabidopsis plants using the Spectrum Plant Total RNA kit (Sigma-Aldrich). cDNA was prepared using oligo(dT) primer and a RevertAid first-strand cDNA synthesis kit (Thermo Fisher Scientific). For quantitative PCR (qPCR), reactions were prepared with a DyNAmo Color-Flash SYBR Green qPCR Kit (Thermo Fisher Scientific) and recorded in a C100 Touch PCR Cycler containing the CFX96 Real-Time System (Bio-Rad). The CFX Manager software (Bio-Rad) was used for data analysis. Primer pairs used in this study are listed in Supplementary Table S1. Mass standards for each gene were used to calculate absolute gene expression, which was normalized to the expression of the *EF1Ba* reference gene. The gene expression was assayed on three independent replicates, averaged and SD was calculated.

### Histochemical staining of ROS

To compare H_2_O_2_ accumulation in seedlings grown under Fe-sufficient or -deficient conditions, 3,3′-diaminobenzidine (DAB; Sigma-Aldrich) staining was performed. After a short vacuum infiltration (5 min, –400 mbar), seedlings were incubated for 1 h in the dark under gentle agitation in DAB solution (1 mg ml^–1^ DAB in 50 mM MES buffer, pH 6.5). Samples were rinsed once with ddH_2_O. EtOH (80%) was added and seedlings were heated to 80 °C for 20 min. Staining was documented on an Axio Imager.M2 microscope (Zeiss).

The distribution of H_2_O_2_ and O_2_˙ ^−^ in roots was visualized by co-staining using the fluorescent dyes 3′-*O*-acetyl-6′-*O*-pentafluoroben-zenesulfonyl-2′,7′-difluorofluorescein (BES-H2O2-Ac, Wako Pure Chemical Corporation) and dihydroethidium (DHE; Sigma-Aldrich). Seedlings grown under Fe-sufficient (50 mM FeNaEDTA, +Fe) or -deficient (0 mM FeNaEDTA, –Fe) conditions for 10 d were incubated for 30 min in the dark at room temperature in liquid half-strength Hoagland medium containing 50 µM BES-H_2_O_2_-Ac and 10 µM DHE.

### Fluorescence microscopy and image analysis

Images of BES-H_2_O_2_-Ac- and DHE-stained roots were taken immediately after the 30 min incubation. For imaging, an Axio Imager.M2 microscope (Zeiss) was used. BES-H_2_O_2_-Ac dye was visualized using Filter set 38 [HE eGFP shift free (E) (EX BP 470/40, BS FT 495, EM BP 525/50)] and DHE using Filter set 43 [HE Cy 3 shift free (E) (EX BP 550/25, BS FT 570, EM BP 605/70)]. All roots were pictured at ×20 magnification in tiles mode, and single images were stitched to produce a composite image of the root. The same imaging settings were employed for all genotypes and replicates.

Images obtained from fluorescent ROS staining were imported into ImageJ (http://rsbweb.nih.gov/ij/) for further analysis. To mark the region of interest (ROI) for signal intensity measurement, a segmented line, synchronized for DHE and BES-H_2_O_2_-Ac channels, was superimposed onto the root. Absolute signal intensity was measured and values were imported into Microsoft Excel. Signal intensity of both channels was normalized to the maximum signal intensity measured for BES-H_2_O_2_-Ac to ensure correct representation of the DHE to BES-H_2_O_2_-Ac signal ratio. This maximum value was set to 100 and all other values are plotted relative to it. Relative signal intensity plots represent the signal either along the root or in virtual cross-sections across three zones. Each plot is an average of three biological replicates with their respective SD.

### Statistical analysis

Data were analyzed using one-way ANOVA followed by Fisher’s least statistical difference post-hoc test in the SPSS Statistics software (IBM). Statistically significant differences were estimated based on *P*-values (*P*<0.05).

## Results

### Root H_2_O_2_ content increases under prolonged Fe limitation

**Fig. 1.**
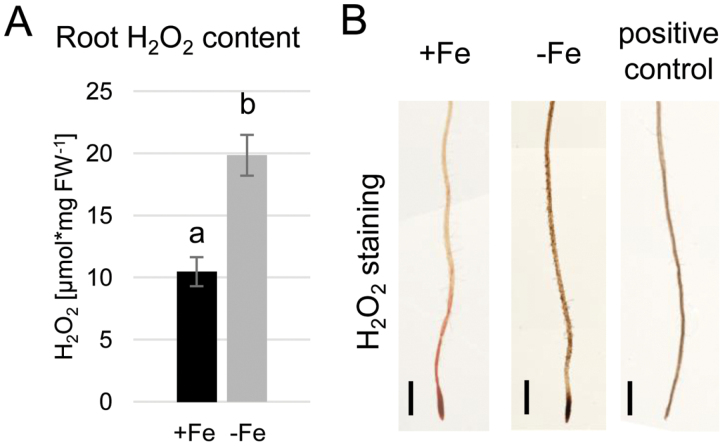
Root H_2_O_2_ content is increased under prolonged Fe deficiency. (A) Quantitative H_2_O_2_ measurement in wild-type roots grown for 10 d under sufficient (+Fe) or deficient (–Fe) Fe supply (*n*=3). Bars represent mean values ±SD. Different letters indicate statistically significant differences (*P*<0.05). (B) Histochemical H_2_O_2_ staining of wild-type roots grown for 10 d under +Fe or –Fe. Positive control: seedling damaged by wounding to induce H_2_O_2_ accumulation. Size bars=0.5 cm.

Increased H_2_O_2_ content in the root was previously shown to influence responses to prolonged Fe deficiency (Le *et al.*, 2016). We aimed to understand whether this increase affected the whole root equally or whether the observation could be linked to certain root zones, such as those responsible for the acquisition of Fe. First, we performed a quantitative measurement of endogenous H_2_O_2_ in plants grown for 10 d under either Fe-sufficient or Fe-deficient conditions. As quantified in Fig. 1A, the overall H_2_O_2_ content of Fe-deficient plants was significantly increased compared with the control Fe condition. We next performed DAB staining for visualizing H_2_O_2_ in the root (Fig. 1B). A general increase in staining could be observed in Fe-deficient roots, consistent with the quantitative data. To confirm the specificity of the staining, cotyledons of a control plant were manually wounded with forceps. The elicited stress response caused an increase in H_2_O_2_ levels in the whole seedling, resulting in strong DAB staining (Fig. 1B). The data suggested a general increase in H_2_O_2_ content throughout the root under prolonged Fe deficiency. However, due to the low dynamic range of the staining method, it did not provide sufficient detail on potential fine differences between different root zones.

### ROS distribution depends on Fe availability and the plant’ capacity to take up Fe

**Fig. 2.**
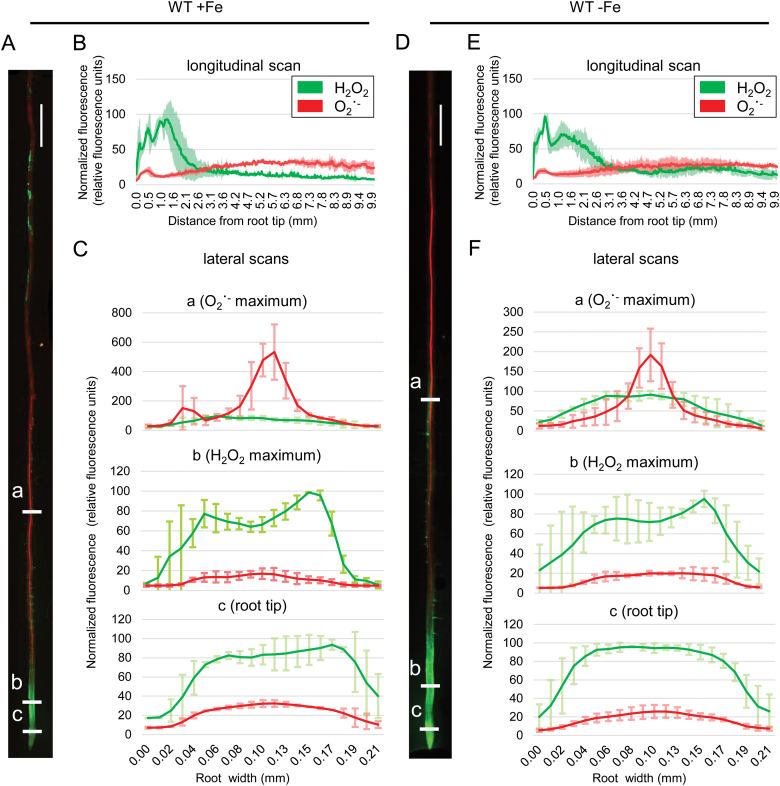
Histochemical H_2_O_2_ and O_2_˙ ^−^ staining in wild-type roots grown for 10 d under sufficient (+Fe) or deficient (–Fe) Fe supply. (A) Staining example of a root grown under +Fe. (B) Longitudinal signal intensity scan of roots grown under +Fe, ±SD. (C) Lateral signal intensity scans at three different positions, indicated as ‘a’, ‘b’, and ‘c’ in (A), of roots grown under +Fe. (D) Staining example of a root grown under –Fe. (E) Longitudinal signal intensity scan of roots grown under –Fe. (F) Lateral signal intensity scans at three different positions, indicated as ‘a’, ‘b’, and ‘c’ in (D), of roots grown under –Fe. All graphs represent averaged data from five roots, ±SD. Size bars in (A) and (D): 0.5 cm.

Previously, it was shown that the ratio between H_2_O_2_ and O_2_˙ ^−^ along the root is important for defining the differentiation state of the root cells (Tsukagoshi *et al.*, 2010; Reyt *et al.*, 2015). Therefore, in order to obtain a detailed map of ROS distribution within the root under prolonged Fe deficiency, we performed a dual histochemical staining for H_2_O_2_ and superoxide (O_2_˙ ^−^; Fig. 2), using BES-H_2_O_2_-Ac and DHE, respectively. This allows the distinction of the tissue-level localization of the two ROS species simultaneously and *in vivo*. In wild-type Fe-sufficient roots, we observed strong H_2_O_2_ staining in the root tip and a gradual decrease of signal towards the root base. In the differentiation zone, the signal was observed in both the central cylinder and the epidermis (Fig. 2A–C, green channel). Signal quantification confirmed this, showing that the fluorescent intensity peaked in the transition zone between the meristem and the elongation zone (Fig. 2A, B, green channel). This observation is consistent with previous reports (Tsukagoshi *et al.*, 2010; Reyt *et al.*, 2015). The O_2_˙ ^−^ pattern, on the other hand, differed greatly along the root. We observed a small maximum at the tip of the meristem above the stem cell niche. However, the majority of the signal was present in the differentiation zone, especially in the central cylinder (Fig. 2A–C, red channel). Prolonged Fe deficiency resulted in the expansion of the H_2_O_2_ domain within the meristem together with a marked increase in signal intensity throughout the root (Fig. 2D–F, green channel), consistent with our DAB staining and the quantitative H_2_O_2_ measurement.

**Fig. 3.**
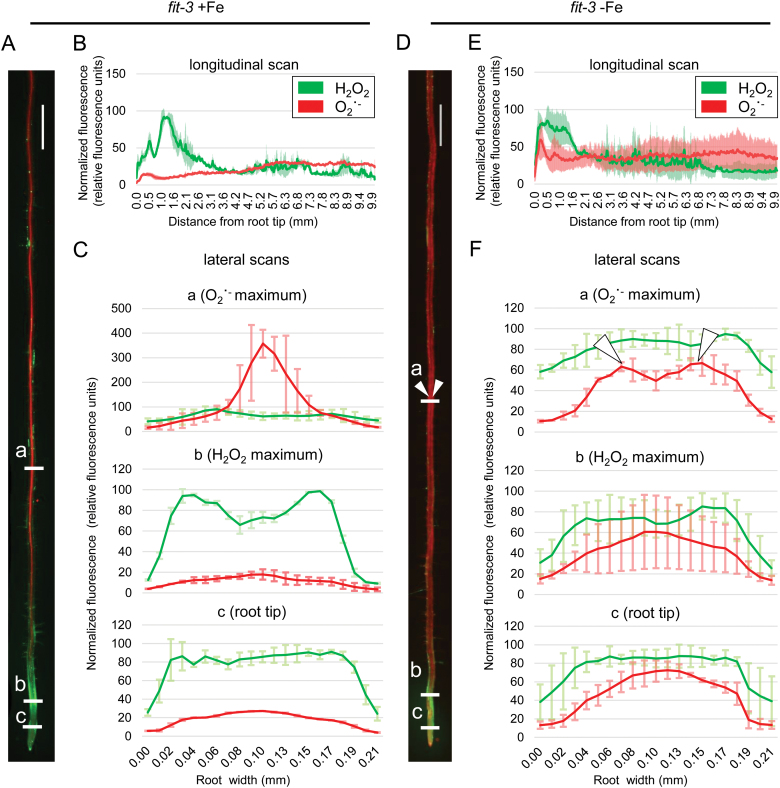
Histochemical H_2_O_2_ and O_2_˙ ^−^ staining in *fit-3* roots grown for 10 d under sufficient (+Fe) or deficient (–Fe) Fe supply. (A) Staining example of a root grown under +Fe. (B) Longitudinal signal intensity scan of roots grown under +Fe supply, ±SD. (C) Lateral signal intensity scans at three different positions, indicated as ‘a’, ‘b’, and ‘c’ in (A), of roots grown under +Fe. (D) Staining example of a root grown under –Fe. White arrowheads highlight the predominant O_2_˙ ^−^ signal in the peripheral root zones. (E) Longitudinal signal intensity scan of roots grown under –Fe. (F) Lateral signal intensity scans at three different positions, indicated as ‘a’, ‘b’, and ‘c’ in (D), of roots grown under –Fe. White arrowheads in the top graph highlight the predominant O_2_˙ ^−^ signal in the peripheral root zones and correspond to the arrowheads in (D). All graphs represent averaged data from five roots, ±SD. Size bars in (A) and (D): 0.5 cm.

The FIT loss-of-function mutant *fit-3* (Jakoby *et al.*, 2004) was previously reported to lack prolonged Fe deficiency-induced H_2_O_2_ accumulation, suggesting that ROS homeostasis might be under the control of FIT (Le *et al.*, 2016). Roots of *fit-3* grown under control Fe conditions showed distribution patterns for both H_2_O_2_ and O_2_˙ ^−^ comparable with those of the wild type (Fig. 3A–C). As expected, under Fe deficiency, the H_2_O_2_ pattern remained unchanged compared with the sufficient Fe condition (Fig. 3D–F). A prominent effect occurred under Fe deficiency, where the lateral distribution of the O_2_˙ ^−^ signal shifted from the central cylinder and was instead very prominent in the *fit-3* root periphery (Fig. 3D, F). Our findings suggest that in the absence of FIT, the H_2_O_2_ to O_2_˙ ^−^ ratio cannot adjust to the prolonged Fe deficiency conditions, implying a major role for the Fe deficiency regulator FIT in ROS signaling as part of the Fe deficiency response cascade.

### FIT is required for the transcriptional regulation of H_2_O_2_ dismutases in roots

One possible explanation for the observed H_2_O_2_ localization in the root under Fe deficiency is differential activity of H_2_O_2_-metabolizing enzymes. FIT may affect the formation of ROS at the transcriptional level, for example through controlling gene expression of catalases, enzymes that eliminate H_2_O_2_.

We analyzed the expression of three catalase gene isoforms, *CAT1*, *CAT2*, and *CAT3*, due to their very prominent activity and the dependence of their products on the Fe state of the plant, being Fe cofactor-dependent enzymes (Nicholls *et al.*, 2000). For identifying potential Fe and FIT transcriptional targets among the three catalase genes, we reasoned that the candidate regulatory gene should be up-regulated under Fe deficiency and behave oppositely in the absence of functional FIT.

**Fig. 4.**
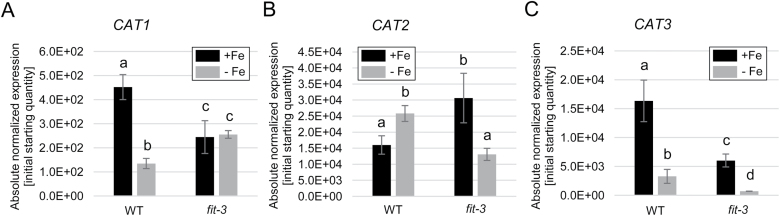
Expression of the genes encoding three Arabidopsis catalase isoforms, (A) *CAT1*, (B) *CAT2*, and (C) *CAT3*, in roots of 10-day-old wild-type and *fit-3* mutant plants grown under sufficient (+Fe) or deficient (–Fe) Fe supply (*n*=3). Bars represent mean values ±SD. Different letters indicate statistically significant differences (*P*<0.05).

Gene expression profiling revealed that *CAT1* underwent down-regulation under prolonged Fe deficiency in the wild type, whereas, in the *fit-3* mutant, a reduced and non-Fe-responsive expression was observed (Fig. 4A). *CAT2* showed a significant up-regulation under prolonged Fe deficiency in the wild type. Interestingly, in a FIT loss-of-function situation, its regulation was exactly opposite to that observed in the wild type (Fig. 4B), suggesting a strong dependence of *CAT2* expression on the function of FIT. Furthermore, *CAT2* abundance under low Fe supply is most probably a compensatory mechanism to counteract excess H_2_O_2_ levels, hence preventing toxic H_2_O_2_ effects. *CAT3*, similarly to *CAT1*, was down-regulated by prolonged low Fe availability in the wild type. (Fig. 4C) This tendency remained in the *fit-3* mutant; however, in the absence of *FIT*, the overall expression of the gene was strongly reduced (Fig. 4C).

The expression analysis showed that all three catalase-encoding genes were dynamically regulated in response to prolonged Fe deprivation and their expression depended to a different extent on the presence of the central Fe acquisition transcriptional regulator FIT. The above-described *CAT2* expression changes under prolonged Fe deficiency suggested that the CAT2 protein might play a role in modulating H_2_O_2_ levels in these growth conditions.

### Perturbance of intracellular H_2_O_2_ homeostasis compromises root Fe acquisition

**Fig. 5.**
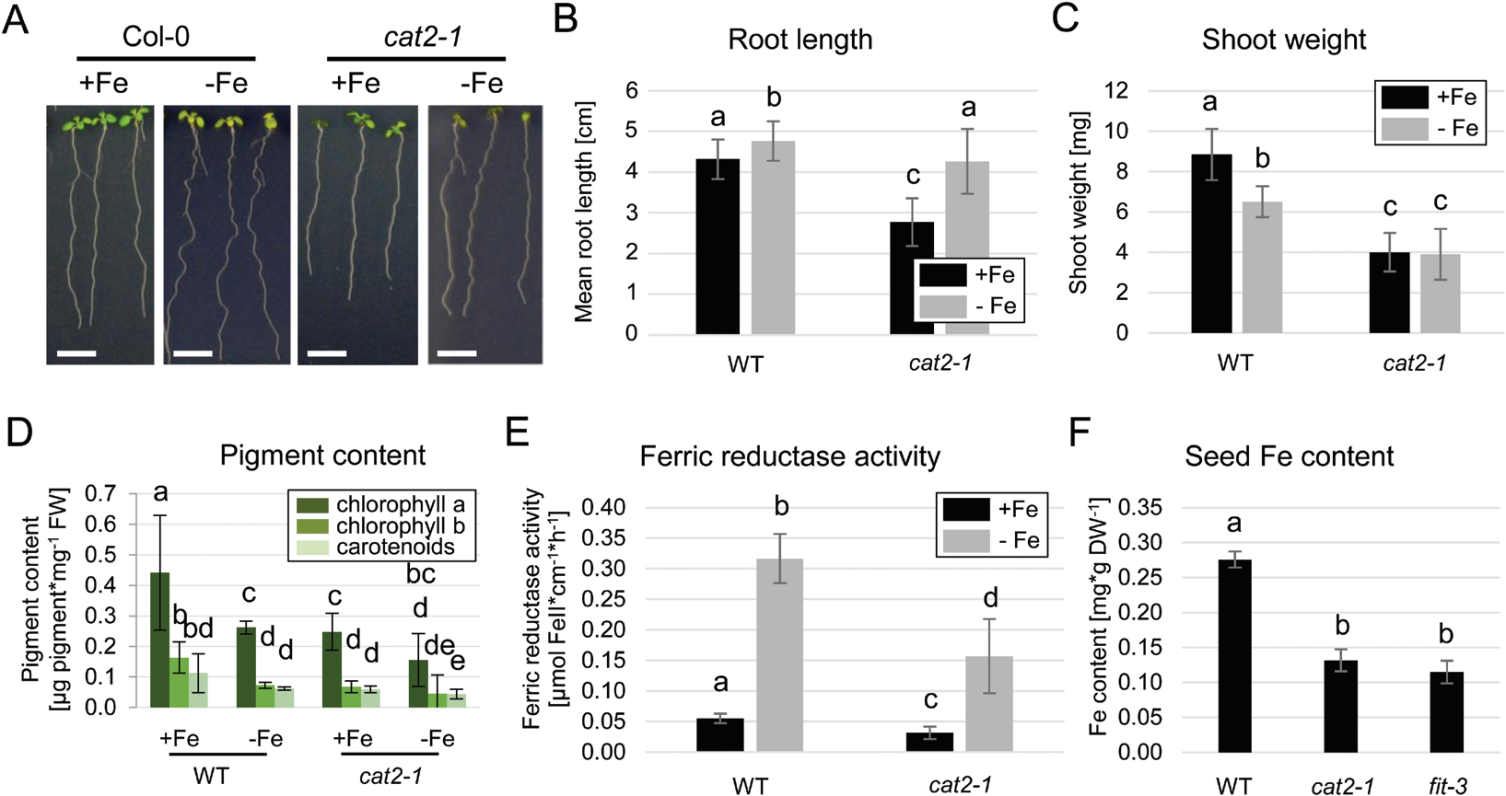
Fe-related phenotypes of *cat2-1* mutant plants. (A) Representative images of wild-type and *cat2-1* plants grown for 10 d under sufficient (+Fe) or deficient (–Fe) Fe supply. Size bars=1 cm. (B) Root length quantification (*n*=27–32). (C) Shoot weight quantification (*n*=6). (D) Photosynthetic pigment quantification (*n*=6). (E) Ferric reductase activity (*n*=6). (F) Seed Fe content of soil-grown wild-type, *cat2-1*, and *fit-3* plants (*n*=3). (B–E) Bars represent mean values ±SD. Different letters indicate statistically significant differences (*P*<0.05).

Our gene expression analysis revealed CAT2 as a potential FIT-dependent regulator of H_2_O_2_ levels under prolonged Fe deficiency. To understand how CAT2 participates in the regulation of Fe deficiency responses, we investigated the Fe response in the previously characterized CAT2 loss-of-function mutant *cat2-1* (Bueso *et al.*, 2007; Queval *et al.*, 2007). In wild-type plants, Fe deficiency resulted in the well-documented elongation of the primary root (Gruber *et al.*, 2013; Ivanov *et al.*, 2014; Gratz *et al.*, 2019), which was also the case for the *cat2-1* mutant. However, the length of the primary *cat2-1* root was reduced by 13% compared with that of the wild type. Under sufficient Fe supply, *cat2-1* root length was reduced even more, by 35% compared with the wild type (Fig. 5A, B). This could be due to the combined deleterious effects of reduced H_2_O_2_-scavenging capacity of the mutant and the available Fe.

Although *cat2-1* roots were able to elongate under prolonged Fe deficiency, the penalty on the shoot weight of the mutant was strong under both Fe supply conditions (Fig. 5C), probably due to the photorespiratory phenotype of *cat2-1*, and consistent with the documented major role of CAT2 in controlling ROS homeostasis in leaves (Mhamdi *et al.*, 2010).

The degradation of photosynthetic pigments is a well-documented effect under Fe deficiency (Ivanov *et al.*, 2012), and we could observe it in our test system (Fig. 5D). In *cat2-1* plants, the overall levels of chlorophyll and carotenoids were significantly lower than in the wild type and were further reduced under prolonged Fe limitation (Fig. 5D). Root surface ferric reductase activity was tested as an indicator for the plant capacity to take up Fe. Activity levels were reduced in *cat2-1* even though the ferric reductase responded to Fe limitation (Fig. 5E). We then grew plants on soil and measured the Fe content in their seeds, as a direct consequence of the plant Fe status (Fig. 5F). As confirmation to our observations so far, *cat2-1* seeds contained low levels of Fe, comparable with the strongly Fe-deficient *fit-3* mutant (Fig. 5F). Overall, we characterized *cat2-1* loss-of-function plants as strongly Fe deficient and unable to induce Fe uptake to counteract Fe deficiency.

### CAT2 participates in modulating root ROS accumulation under prolonged Fe deficiency

**Fig. 6.**
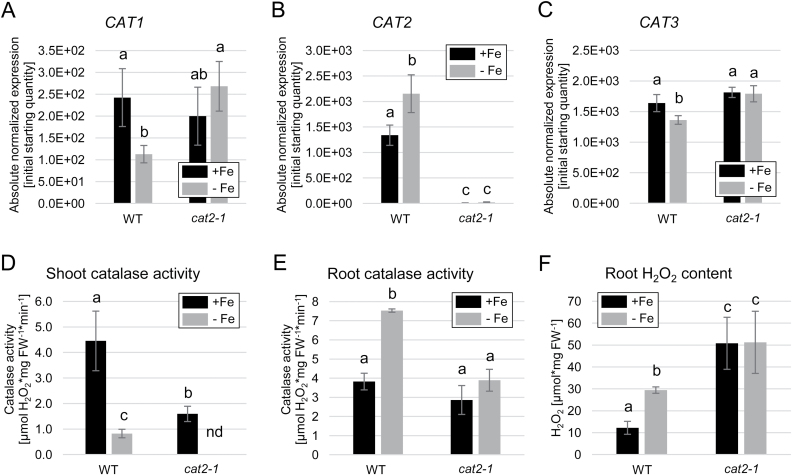
Role of CAT2 in root catalase activity and H_2_O_2_ accumulation under prolonged Fe deficiency. (A–C) Gene expression of (A) *CAT1*, (B) *CAT2*, and (C) *CAT3* in wild-type and *cat2-1* plants grown for 10 d under sufficient (+Fe) or deficient (–Fe) Fe supply (*n*=3). (D) Shoot catalase activity in the wild type and *cat2-1* (*n*=3). (E) Root catalase activity in the wild type and *cat2-1* (*n*=3). (F) Root H_2_O_2_ content in the wild type and *cat2-1* (*n*=3). Bars represent mean values ±SD. Different letters indicate statistically significant differences (*P*<0.05).

The impaired Fe mobilization under prolonged Fe deficiency in the *cat2-1* mutant suggests that enhanced H_2_O_2_ was responsible for this effect. In order to analyze the significance of CAT2 for ROS accumulation under prolonged Fe deficiency, we first evaluated the expression of all *CAT* genes in *cat2-1* plants (Fig. 6A–C). As expected, *CAT1* expression was reduced under Fe deficiency in the wild type, while the levels in *cat2-1* remained comparable with the wild-type sufficient Fe condition. As expected, *CAT2* transcript was not detected in the *cat2-1* mutant, and *CAT3* showed a similar expression tendency to *CAT1* (Fig. 6A–C). This indicated that the expression of *CAT1* and *CAT3* was higher than usual in the absence of CAT2 under prolonged Fe deficiency and pointed towards potential compensatory regulation of *CAT1* and *CAT3* for the missing CAT2, most probably in response to enhanced ROS levels. To test the effect of this potential compensation, we performed a total catalase activity measurement in shoots and roots of wild-type and *cat2-1* plants. In shoots, lack of CAT2 led to a dramatic reduction in overall shoot catalase activity under both sufficient Fe and prolonged Fe deficiency (Fig. 6D), consistent with available data (Queval *et al.*, 2007). In roots, the effect was also significant, but less prominent (Fig. 6E). Fe-deficient wild-type root extracts showed enhanced catalase activity compared with those from sufficient Fe-grown roots. Compared with the wild type, in *cat2-1*, the catalase activity under sufficient Fe was similar; however, it was significantly reduced under prolonged Fe deficiency (Fig. 6E). At the same time, the *fit-3* mutant showed strongly reduced catalase activity (Supplementary Fig. S1), suggesting that the presence of a functional FIT positively influences the catalase function. We then tested the net outcome of these effects by measuring the total H_2_O_2_ content of the root. As already shown, prolonged Fe deficiency led to a marked increase in H_2_O_2_ levels in the wild type (Fig. 6F). In *cat2-1*, the overall H_2_O_2_ accumulation was increased compared with the wild type under both conditions, indicating a prominent role for CAT2 in maintaining the ROS balance in roots (Fig. 6F). The data suggest that despite potential compensation from CAT1 and CAT3, CAT2 is the isoform utilized by roots to control the levels of intracellular H_2_O_2_ under prolonged Fe limitation. One must note that additional ROS-scavenging enzymes might play a role in this process, explaining, for example, the pronounced H_2_O_2_ increase in *cat2-1* under sufficient Fe supply.

**Fig. 7.**
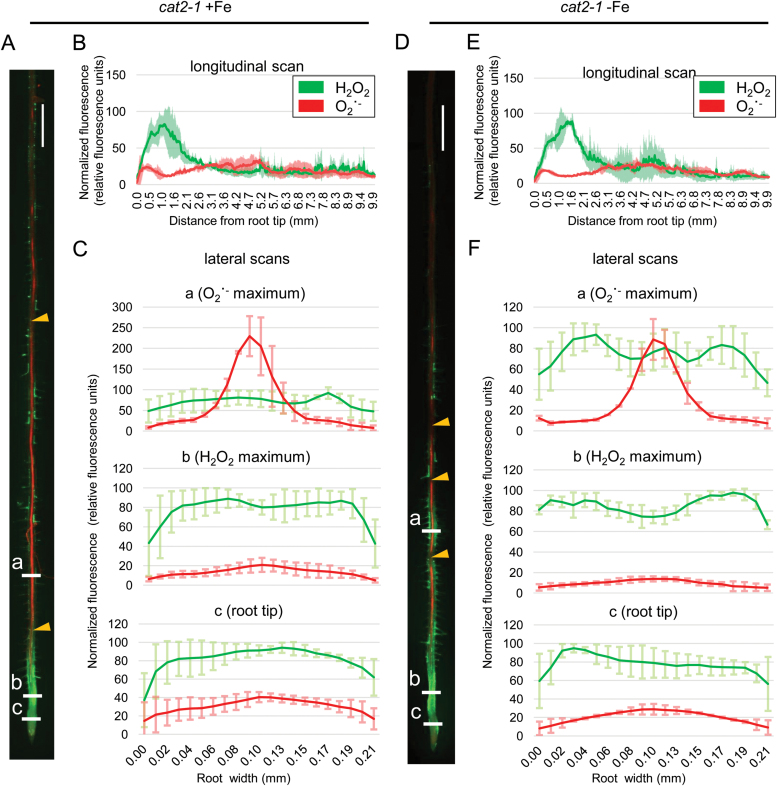
Histochemical H_2_O_2_ and O_2_˙ ^−^ staining in *cat2-1* roots grown for 10 d under sufficient (+Fe) or deficient (–Fe) Fe supply. (A) Staining example of a root grown under +Fe. (B) Longitudinal signal intensity scan of roots grown under +Fe, ±SD. (C) Lateral signal intensity scans at three different positions, indicated as ‘a’, ‘b’, and ‘c’ in (A), of roots grown under +Fe. (D) Staining example of a root grown under –Fe. (E) Longitudinal signal intensity scan of roots grown under –Fe. (F) Lateral signal intensity scans at three different positions, indicated as ‘a’, ‘b’, and ‘c’ in (D), of roots grown under –Fe. All graphs represent averaged data from five roots ±SD. Yellow arrowheads in (A) and (D) indicate regions along the central cylinder lacking O_2_˙ ^−^ signal. Size bars in (A) and (D): 0.5 cm.

To estimate the effect of CAT2 at the organ level, we stained *cat2-1* roots for H_2_O_2_ and O_2_˙ ^−^ (Fig. 7). Compared with the wild type (Fig. 2), *cat2-1* showed a similar staining pattern, with a H_2_O_2_ peak in the transition zone and a small O_2_˙ ^−^ peak in the meristem; however, we observed major changes in the differentiation zone (Fig. 7A–C, green channel). Here, strong H_2_O_2_ signal was observed in the epidermis, particularly prominent in some but not all root hair cells. O_2_˙ ^−^ signal was confined mainly in the central cylinder; however, along the length of the root, we observed zones without staining (Fig. 7A, B, red channel). These zones lacking O_2_˙ ^−^ were more prominent and more frequent under prolonged Fe deficiency, where H_2_O_2_ staining was also strong, especially in root hair cells close to the root tip (Fig. 7D–F). The overall picture in the absence of CAT2 showed a disturbed ROS homeostasis, with over-pronounced H_2_O_2_ accumulation in the differentiation zone across the root, masking O_2_˙ ^−^ levels, and hence resulting in a disturbed balance between the ROS species under prolonged Fe deficiency. These enhanced H_2_O_2_ levels could provoke stress response signals due to ROS toxicity, overpowering the signaling role of H_2_O_2_ in activating the Fe deficiency response network.

### Coordination of transcriptional Fe homeostasis response is compromised in the absence of a functional *CAT2* gene

**Fig. 8.**
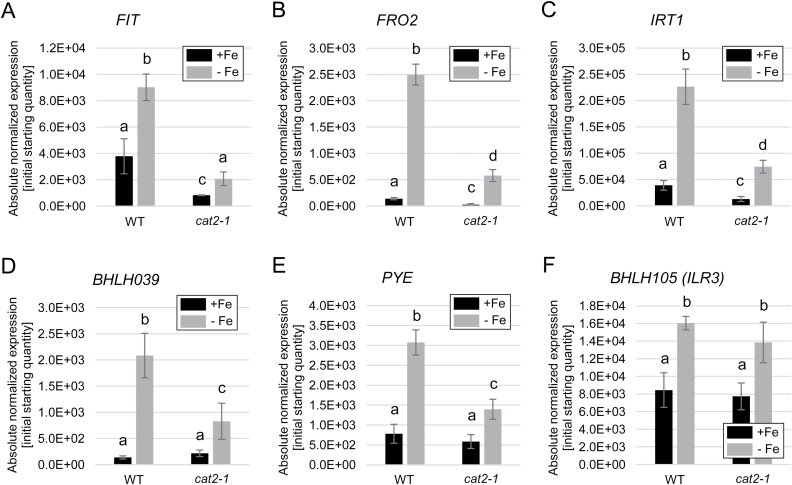
Regulation of Fe acquisition and homeostasis genes in response to increased H_2_O_2_ caused by the absence of CAT2. (A–F) Gene expression of (A) *FIT*, (B) *FRO2*, (C) *IRT1*, (D) *BHLH039*, (E) *PYE*, and (F) *BHLH105* (*ILR3*) in wild-type and *cat2-1* plants grown for 10 d under sufficient (+Fe) or deficient (–Fe) Fe supply (*n*=3). Bars represent mean values, ±SD. Different letters indicate statistically significant differences (*P*<0.05).

Based on the observed phenotypes of deregulated Fe mobilization in *cat2-1* loss-of-function plants, we next aimed to uncover the transcriptional regulation that underlies this susceptibility to prolonged Fe deficiency. We first investigated the expression of the Fe uptake-related genes that are under the control of FIT, namely *FIT* itself, *FRO2*, and *IRT1* (Fig. 8A–C). In the wild type, all three genes performed as previously reported, with expression up-regulated under Fe deficiency. However, in the *cat2-1* mutant, the expression was markedly decreased in both Fe conditions relative to the wild type (Fig. 8A–C). This suggests that together with the increased H_2_O_2_ levels in the absence of CAT2, Fe uptake genes undergo repression or are hardly expressed, consistent with the reported effects of external H_2_O_2_ application and the action of ZAT12 as a FIT inhibitor (Le *et al.*, 2016). The ROS-responsive *ZAT12* gene showed strong up-regulation in *cat2-1*, as expected because of enhanced H_2_O_2_ levels in this mutant (Supplementary Fig. S2). We then tested the expression of the *BHLH039* gene encoding a FIT partner and positive Fe deficiency response regulator (Wang *et al.*, 2007; Yuan *et al.*, 2008; Wang *et al.*, 2013). *BHLH039* transcriptional up-regulation does not require FIT protein but, instead, it is directly coupled to an Fe deficiency signaling cascade upstream of FIT, mediated by bHLH transcription factors of the subgroups IVb and IVc, such as URI and ILR3 (Samira *et al.*, 2018; Kim *et al.*, 2019; Tissot *et al.*, 2019; Lei *et al.*, 2020; Gao *et al.*, 2020*a*). In the absence of FIT, the *BHLH039* gene is strongly up-regulated; however, in the absence of ZAT12, *BHLH039* expression is reduced. These expression patterns can be explained by the different Fe levels, caused by different FIT activity, in wild-type and *zat12* mutant plants (Le *et al.*, 2016). Interestingly, *BHLH039* expression in *cat2-1* was strongly reduced (Fig. 8D), similarly to *FIT*. This suggested that the link coordinating the expression of the two genes is dependent on the function of CAT2. The genes encoding the closest bHLH039 homologs, *BHLH038*, *BHLH100*, and *BHLH101*, were similarly down-regulated in *cat2-1* in comparison with the wild type (SupplementaryFig. S3A–C). Thus, the upstream bHLH signaling cascade involving the bHLH subgroup IVb and IVc transcription factors is also dependent on ROS regulation.

This was confirmed when we tested additional regulatory genes, encoding the Fe homeostasis regulator PYE (Long *et al.*, 2010), from subgroup IVb, and the upstream regulators ILR3 and BHLH104 (Zhang *et al.*, 2015), from subgroup IVc. *PYE* gene expression is co-regulated with that of *BHLH039*, and, similarly to *BHLH039*, the expression of *PYE* was reduced in the *cat2-1* mutant (Fig. 8E), confirming that the upstream regulation is affected by cellular H_2_O_2_ levels. In contrast, *ILR3* (Fig. 8F) and *BHLH104* (Supplementary Fig. S3D) expression remained unaffected in *cat2-1* compared with the wild type, suggesting that a H_2_O_2_-dependent post-translational event inhibits the activity of ILR3 and its homologs in response to prolonged Fe deficiency.

## Discussion

**Fig. 9.**
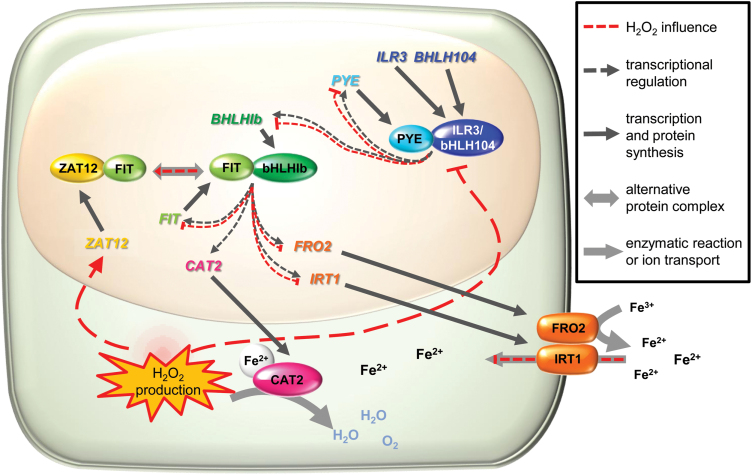
Proposed model for the role of FIT, CAT2, and H_2_O_2_ in the regulation of Fe acquisition and homeostasis under prolonged Fe deficiency. Subgroup IVc proteins ILR3 and bHLH104, together with other transcription factors (not depicted), form a complex with PYE and regulate the expression of downstream Fe homeostasis genes (dark dotted arrows), including *PYE* itself and subgroup Ib *BHLH*genes (*BHLH038*, *BHLH039*, *BHLH100*, and *BHLH101*). Subgroup Ib bHLHs interact with FIT, a complex which positively regulates FIT, Fe acquisition genes, such as *FRO2* and *IRT1*, and *CAT2*. CAT2 protein is a heme-containing H_2_O_2_ dismutating enzyme, which requires Fe, imported with the help of FRO2 ferric reductase and the transporter IRT1. Under prolonged Fe deficiency, H_2_O_2_ production is increased and acts in two directions (red dotted lines). First, high H_2_O_2_ levels negatively influence the positive regulation by PYE–ILR3/bHLH104. Thus, transcription of *PYE* and subgroup Ib *BHLH* genes is down-regulated. This depletion of subgroup Ib bHLH proteins is the first factor that negatively influences the formation of the positive Fe acquisition transcriptional complex FIT–bHLH Ib and results in attenuation of *FRO2* and *IRT1* expression, and therefore Fe import. The second effect of H_2_O_2_ accumulation is the activation of the ROS-responsive transcription factor ZAT12. ZAT12 can interact with FIT, forming an inactive FIT complex. This is the second factor that prevents the formation of the FIT–bHLH Ib positive regulatory complex. The role of CAT2 would be to limit the H_2_O_2_ levels by dismutating the excess H_2_O_2_ to balance the efficiency at which the transcriptional regulatory cascade functions.

ROS signaling is one of the key determinants underlying plant environmental responses (He *et al.*, 2018). Among ROS, H_2_O_2_ is directly involved in the regulation of responses to prolonged Fe deficiency through promoting the expression of the transcription factor ZAT12 and regulating its stability (Brumbarova *et al.*, 2016*b*; Le *et al.*, 2016). In this study, we demonstrate that the accumulation of H_2_O_2_ in Arabidopsis roots under prolonged Fe deficiency is dependent on the activity of CAT2 (Figs 6F, 7). With the help of a CAT2 loss-of-function mutant, we were able to demonstrate that H_2_O_2_-dependent regulation affects not only the Fe uptake-related FIT target genes (Fig. 8A–C) but also the genes encoding the transcription factors from subgroup Ib (bHLH038, bHLH039, bHLH100, and bHLH101) and PYE (Fig. 8D, E; Supplementary Fig. S3A–C). The genes encoding the upstream regulators ILR3 and bHLH104 are themselves not affected by the increased H_2_O_2_ levels in the *cat2-1* mutant (Fig. 8F; Supplementary Fig. S3D). However, ILR3 and bHLH104 protein activity is affected, a fact that points in the direction of an H_2_O_2_-dependent post-translational regulation of these proteins (Fig. 9).

Our data suggest that the role of CAT2 in this complex regulatory interplay most probably is to control the upper limit of available H_2_O_2_ under prolonged Fe deficiency. Under this condition, an as yet unidentified H_2_O_2_ source is activated and CAT2 activity is enhanced to maintain a balance of higher than usual cellular H_2_O_2_ levels. The data suggest that even though in roots CAT2 shares activity with the two other isoforms, CAT1 and CAT3, it is still the predominant catalase isoform determining H_2_O_2_ degradation under prolonged Fe deficiency. This is supported by the fact that *cat2-1* plants exhibit decreased catalase activity and increased H_2_O_2_ cellular levels compared with the wild type under these conditions (Fig. 6E, F). In terms of gene expression, previous studies reported no compensatory effect of *CAT1* and *CAT3* expression in the absence of CAT2 in leaves (Queval *et al.*, 2007). Under Fe deficiency, however, the expression of *CAT1* and *CAT3* was increased in roots of *cat2-1* compared with the wild type, probably as an attempt at compensation for the missing CAT2 (Fig. 6A–C).

The obtained data support a model in which CAT2 is deeply integrated in the cellular regulatory events under prolonged Fe limitation (Fig. 9). The *CAT2* gene closely responds to the Fe status of the root and the presence of FIT, suggesting that it is either a direct FIT target or a strongly FIT-dependent gene. Indirectly, through H_2_O_2_ availability and potentially additional players, including FIT, the activity of CAT2 modulates the functionality of the Fe response transcriptional cascade. As the *CAT2* gene is dependent on the function of this cascade, this also functions as a feedback loop for the regulation of CAT2. An additional level of interplay between CAT2 and Fe response comes from the end result of the transcriptional cascade, namely the production of the IRT1 transporter, that provides Fe necessary for the enzymatic activity of CAT2.

An interesting question concerns the localization of the CAT2-derived effects, on both the subcellular and whole-organ level. CAT2 was known as an exclusively peroxisomal protein; however, recent studies clearly demonstrate the presence, function, and significance of catalases outside the peroxisome. Examples are available for additional CAT roles in the cytoplasm and the nucleus (He *et al.*, 2018). It has also been proposed that CATs can relocate in the cell in response to redox signals (Foyer *et al.*, 2020). At this point, it is not possible to speculate if a specific compartment might harbor catalase activity orchestrating the responses to prolonged Fe limitation. At the same time, however, our data clearly suggest the domains within the root where the described regulatory events might take place. In wild-type plants under prolonged Fe deficiency, the H_2_O_2_ level in the differentiation zone of the root was clearly increased, especially in regions where O_2_˙ ^−^, visible in the central cylinder, reached a plateau (Fig. 2D–F). The presence of H_2_O_2_ in this region, which we refer to as the early differentiation zone, is very pronounced in root hair cells, coinciding very well with the reported expression of the principal Fe transporter IRT1 (Barberon *et al.*, 2011; Blum *et al.*, 2014). These observations suggest that the root hair cells of the early differentiation zone are an important location where the described regulatory crosstalk occurs. This is supported by the fact that under prolonged Fe deficiency, *cat2-1* plants show strong H_2_O_2_ signal in root hair cells of this region (Fig. 7D–F), suggesting that in root hair cells the role of CAT2 is to metabolize excess H_2_O_2_ to maintain ROS balance and coordinate Fe responses.

Excess Fe supply is another condition that requires adjustment of the Fe uptake capacity of the plant to avoid Fe toxicity. It was previously demonstrated that the balance between H_2_O_2_ and O_2_˙ ^−^ in the root meristematic and differentiation zones partially mediated the response of the root system architecture to Fe excess (Reyt *et al.*, 2015).

At present, the details of the mechanistic role of H_2_O_2_ in the process remain unclear. *FIT* and its target genes are dependent on the presence of bHLH039. *BHLH39* and *PYE* are co-expressed, and their promoters are up-regulated in response to Fe deficiency by the bHLH subgroup IVc transcription factors (Gao *et al.*, 2019). Subgroup IVc bHLHs ILR3 and bHLH104 are transcriptionally up-regulated by Fe deficiency (Wang *et al.*, 2017; Samira *et al.*, 2018), similarly to FIT and subgroup Ib bHLHs. Very interestingly, however, their gene expression was not affected by ROS in the *cat2-1* mutant (Fig. 8F; Supplementary Fig. S3D), while their protein activity, judged by the expression of their direct target genes *BHLH039* and *PYE* (Fig. 8D, E), was inhibited by ROS in *cat2-1*. Low subgroup Ib transcription factor activity leads to lower FIT activity and Fe acquisition downstream of FIT in *cat2-1* (Figs 5E, F, 8A–C). Our data conform with previous observations from *BHLH039* overexpression in plants (Naranjo-Arcos *et al.*, 2017). It was shown that increased Fe levels due to *BHLH039* overexpression resulted in a down-regulation of all *BHLH* subgroup Ib genes and *PYE*, indicating that the Fe deficiency response cascade acting upstream of these genes was not active. At the same time, *BHLH039*-overexpressing plants had high levels of ROS and, accordingly, elevated *ZAT12* expression along with symptoms of oxidative stress. Here, we show that the presence of ROS, even in the absence of Fe, is responsible for switching off the Fe deficiency response. ZAT12 may be one of the regulators mediating the inhibition through FIT interaction.

Therefore, in our model, we propose that H_2_O_2_ post-translationally inhibits transcriptional regulators, such as ILR3 and bHLH104, and therefore causes the observed downstream transcriptional changes. Redox-dependent conformational changes have been shown to affect protein activity and nuclear localization, for example of NONEXPRESSOR OF PATHOGENESIS-RELATED GENE 1 (NPR1), involved in pathogen response (Mou *et al.*, 2003; Tada *et al.*, 2008). Alternatively, or additionally, redox-sensing mechanisms may involve differential binding to a partner protein, or altered protein stability and DNA binding activity (He *et al.*, 2018). Further studies are needed to determine whether the transcription factor proteins ILR3, bHLH104, or other subgroup IVc proteins or URI are directly controlled by ROS. Their activity is regulated by Fe-dependent post-translational control mechanisms. These include, for example, E3 ligases, determining bHLH transcription factor protein abundance, and their activity might also be ROS controlled (Hindt *et al.*, 2017).

Previous studies have proposed that ROS regulation displays considerable interplay with other compounds, such as reactive nitrogen species (Wang and Chu, 2020), among which nitric oxide has been shown to affect plant responses to Fe limitation (Graziano and Lamattina, 2007; Meiser *et al.*, 2011). Another interesting aspect of ROS is their interplay with calcium (Ca^2+^) signaling (Kollist *et al.*, 2019). The signal relay between ROS and Ca^2+^ in the central cylinder may result in the generation of O_2_˙ ^−^ through the action of RESPIRATORY BURST OXIDASE HOMOLOGS (RBOHs). As this radical is short-lived and is quickly converted to H_2_O_2_ (Jimenez-Quesada *et al*., 2016), it is of great interest as a potential H_2_O_2_ donor for the above-described signaling events. The subcellular origin of H_2_O_2_ production is important, as it may influence the transcriptional outcome (Sewelam *et al.*, 2014). Excess H_2_O_2_ might inhibit the Ca^2+^–ROS cascade and such an inhibition might be the reason for the ‘gaps’ we observed in the O_2_˙ ^−^ staining in the central cylinder of *cat2-1* plants. Due to this, we cannot exclude that disturbed Ca^2+^ signaling might contribute to the Fe response phenotypes of CAT2 loss-of-function plants, as Ca^2+^ is documented to play a role in both transcriptional (Gratz *et al.*, 2019) and post-translational regulation of Fe import (Dubeaux *et al.*, 2018; Khan *et al.*, 2019), targeting FIT and IRT1 activity, respectively.

In summary, here we described a role for CAT2 in modulating the root responses to prolonged Fe deficiency. This is achieved through the regulation of H_2_O_2_ levels in root epidermis cells, where the Fe deficiency-induced increase of generated H_2_O_2_ is counteracted by CAT2 to a level that prevents toxic H_2_O_2_ effects and yet allows the maintenance of sufficient H_2_O_2_ amounts to coordinate the transcriptional Fe response. Through mutual regulation, CAT2 and FIT represent components of a regulatory loop for regulation of plant responses to Fe deficiency, where H_2_O_2_ functions as an adjustable signaling intermediate.

## Supplementary data

The following supplementary data are available at *JXB* online.

Fig. S1. Catalase activity in *fit-3* mutant plants.

Fig. S2. Regulation of the *ZAT12* gene in wild-type and *cat2-1* plants grown for 10 d under sufficient (+Fe) or deficient (–Fe) Fe supply.

Fig. S3. Regulation of Fe acquisition and homeostasis genes in response to increased H_2_O_2_ caused by the absence of CAT2.

Table S1. List of primers used for RT-qPCR.

eraa522_suppl_Supplementary-Figures-S1-S3_and_Table-S1

## Data Availability

Sequence data from this article can be found in the TAIR and GenBank data libraries under accession numbers: *BHLH038* (AT3G56970), *BHLH039* (AT3G56980), *BHLH047* (*PYE*, AT3G47640) *BHLH100* (AT2G41240), *BHLH101* (AT5G04150), *BHLH104* (AT4G14410), *BHLH105* (*ILR3*, AT5G54680), *CAT1* (AT1G20630), *CAT2* (AT4G35090), *CAT3* (AT1G20620), *FIT* (AT2G28160), *FRO2* (AT1G01580), *IRT1* (AT4G19690), and *ZAT12* (AT5G59820). The data supporting the findings of this study are available from the corresponding author, Tzvetina Brumbarova, upon request.
