# Supplementary material for: Reactive oxygen species coordinate the transcriptional responses to iron availability in Arabidopsis
Source: J Exp Bot. 2020 Nov 7;72(6):2181–95. doi: 10.1093/jxb/eraa522 (PMC7966954; doi:10.1093/jxb/eraa522)
Supplement: eraa522_suppl_Supplementary-Figures-S1-S3_and_Table-S1 [file eraa522_suppl_supplementary-figures-s1-s3_and_table-s1.pdf]

Supplemental data

Reactive Oxygen Species coordinate the transcriptional responses to iron availability in Arabidopsis

Claudia von der Mark[1,2,*], Rumen Ivanov[1,*], Monique Eutebach[1], Veronica G. Maurino[3], Petra Bauer[1,4,†] and Tzvetina Brumbarova[1,†]

[1] Institute of Botany, Heinrich Heine University, Universitätsstr. 1, 40225 Düsseldorf, Germany

[2] Group of Plant Vascular Development, Swiss Federal Institute of Technology (ETH) Zurich, 8092 Zurich, Switzerland

[3] Department of Molecular Plant Physiology, Institute of Molecular Physiology and Biotechnology of Plants, University of Bonn, Kirschalle 1, 53115 Bonn, Germany

[4] Cluster of Excellence on Plant Science (CEPLAS), Heinrich Heine University, 40225 Düsseldorf, Germany

[*] These authors contributed equally to this work.

[†] Correspondence: petra.bauer@hhu.de, tzvetina.brumbarova@hhu.de

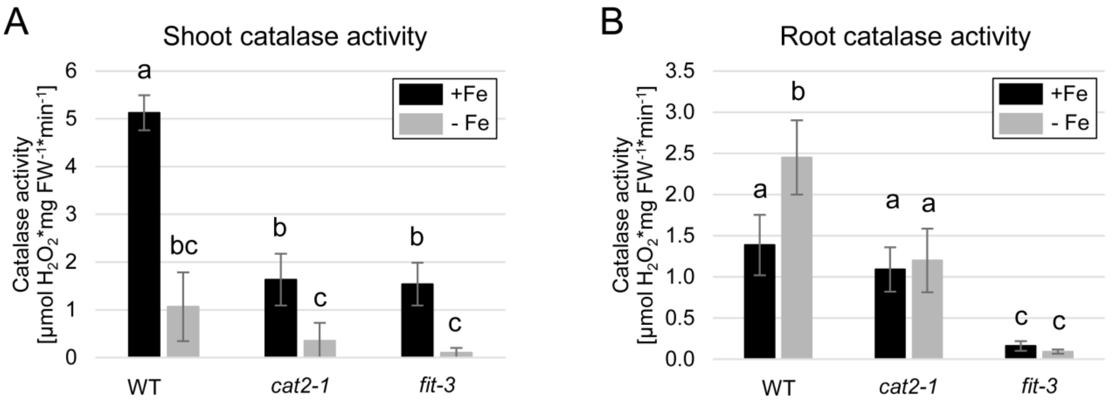

Supplemental Fig. 1. Catalase activity in *fit-3* mutant plants. (A) Shoot catalase activity in wild type, *cat2-1* and *fit-3* plants. (B) Catalase activity in roots of wild type, *cat2-1* and *fit-3*. Plants were grown for 10 days under sufficient (+Fe) or deficient Fe supply (-Fe). Bars represent mean values of three independent experiments ± SD. Different letters indicate statistically significant differences (P < 0.05).

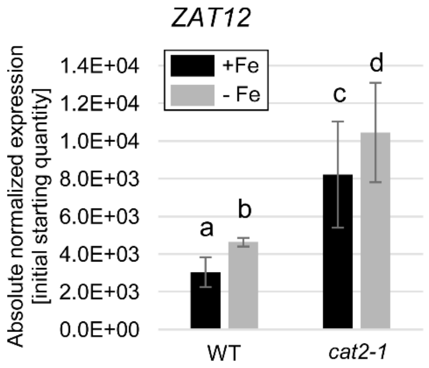

Supplemental Fig. 2. Regulation of the *ZAT12* gene in wild-type and *cat2-1* plants grown for 10 days under sufficient (+Fe) or deficient (-Fe) Fe supply (n = 3). Bars represent mean values, ± SD. Different letters indicate statistically significant differences ($P < 0.05$).

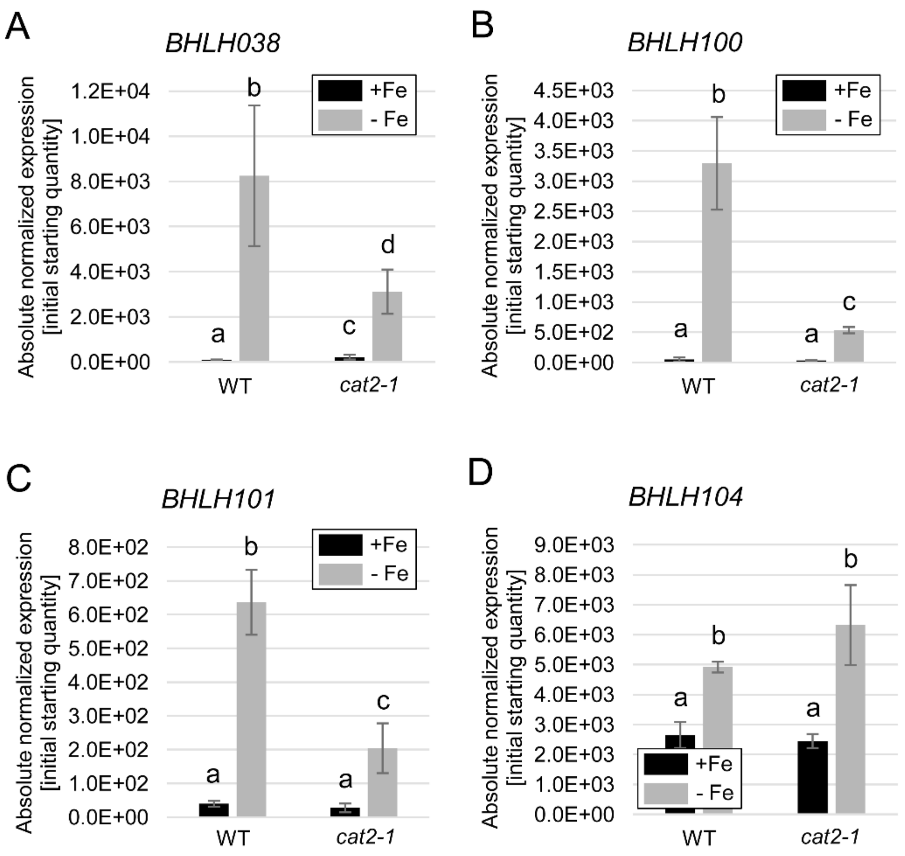

Supplemental Fig. 3. Regulation of Fe acquisition and homeostasis genes in response to increased H_2O_2 caused by the absence of CAT2. (A-D) Gene expression of (A) *BHLH038*, (B) *BHLH100*, (C) *BHLH101* and (D) *BHLH104* in wild-type and *cat2-1* plants grown for 10 days under sufficient (+Fe) or deficient (-Fe) Fe supply (n = 3). Bars represent mean values, ± SD. Different letters indicate statistically significant differences ($P < 0.05$).

Supplemental Table 1. List of primers used for RT-qPCR.

Primer name	Primer sequence (5‘ to 3‘)	Origin
BHLH038 RT-F	AGCAGCAACCAAAGGCG	Wang *et al.* (2007)
BHLH038 RT-R	CCACTTGAAGATGCAAAGTGTAG	Wang *et al.* (2007)
BHLH039 RT-F	GACGGTTTCTCGAAGCTTG	Wang *et al.* (2007)
BHLH039 RT-R	GGTGGCTGCTTAACGTAACAT	Wang *et al.* (2007)
BHLH100 RT-F	AAGTCAGAGGAAGGGGTTACA	Wang *et al.* (2007)
BHLH100 RT-R	GATGCATAGAGTAAAAGAGTCGCT	Wang *et al.* (2007)
BHLH101 RT-F	CAGCTGAGAAACAAAGCAATG	Wang *et al.* (2007)
BHLH101 RT-R	CAGTCTCACTTTGCAATCTCC	Wang *et al.* (2007)
BHLH104 RT-F	GGTTGAGGAGGGAGAAGCTAAATG	This study
BHLH104 RT-R	ACGGATTGCATCATCGAGTATAGC	This study
CAT1 RT-F	AAGTGCTTCATCGGGAAGG	This study
CAT1 RT-R	CTCCGAAAGCGCTTCAAC	This study
CAT2 RT-F	TGCTGGAAACTACCCTGAATGG	This study
CAT2 RT-R	TCAACACCATACGTCCAACAGG	This study
CAT3 RT-F	CCACTTGATGTGACCAAGATCTG	This study
CAT3 RT-R	GTAGATTCCAGGAACCACAAGACC	This study
EFc RT-F	TATGGGATCAAGAAACTCACAAT	Wang *et al.* (2007)
EFc RT-R	CTGGATGTACTCGTTGTTAGGC	Wang *et al.* (2007)
FIT RT-F	GGAGAAGGTGTTGCTCCATC	Wang *et al.* (2007)
FIT RT-R	TCCGGAGAAGGAGAGCTTAG	Wang *et al.* (2007)
FRO2 RT-F	CTTGGTCATCTCCGTGAGC	Wang *et al.* (2007)
FRO2 RT-R	AAGATGTTGGAGATGGACGG	Wang *et al.* (2007)
ILR3 RT-F	GCATGTAGAGAGAAGCAGCGAC	This study
ILR3 RT-R	TGCGGACAGCATCAACCAAG	This study
IRT1 RT-F	AAGCTTTGATCACGGTTGG	Wang *et al.* (2007)
IRT1 RT-R	TTAGGTCCCATGAACTCCG	Wang *et al.* (2007)
PYE RT-F	GTTCCCAGGACTTCCCATTT	This study
PYE RT-R	GTGTCTGGGGATCAGGTTGT	This study
ZAT12 RT-F	GAGTCACAAGAAGCCTAACAACGA	Le *et al.* (2016)
ZAT12 RT-R	AAGCCACTCTCTTCCCACTGCTA	Le *et al.* (2016)

Supplemental References

Le CT, Brumbarova T, Ivanov R, Stoof C, Weber E, Mohrbacher J, Fink-Straube C, Bauer P. 2016. ZINC FINGER OF ARABIDOPSIS THALIANA12 (ZAT12) Interacts with FER-LIKE IRON DEFICIENCY-INDUCED TRANSCRIPTION FACTOR (FIT) Linking Iron Deficiency and Oxidative Stress Responses. Plant Physiol **170**, 540-557.

Wang HY, Klatte M, Jakoby M, Baumlein H, Weisshaar B, Bauer P. 2007. Iron deficiency-mediated stress regulation of four subgroup Ib BHLH genes in *Arabidopsis thaliana*. Planta **226**, 897-908.
